# Intergenerational Patterns of DNA Methylation in *Procambarus clarkii* Following Exposure to Genotoxicants: A Conjugation in Past Simple or Past Continuous?

**DOI:** 10.3390/toxics9110271

**Published:** 2021-10-20

**Authors:** Raquel Marçal, Lola Llorente, Oscar Herrero, Rosario Planelló, Sofia Guilherme, Mário Pacheco

**Affiliations:** 1 Centre for Environmental and Marine Studies (CESAM), Department of Biology, Santiago University Campus, University of Aveiro, 3810-193 Aveiro, Portugal; armarcal@ua.pt (R.M.); sofia.g.guilherme@ua.pt (S.G.); 2 Biology and Environmental Toxicology Group, Faculty of Sciences, Universidad Nacional de Educación a Distancia (UNED), Paseo de la Senda del Rey 9, 28040 Madrid, Spain; lolallorente@ccia.uned.es (L.L.); oscar.herrero@ccia.uned.es (O.H.); rplanello@ccia.uned.es (R.P.)

**Keywords:** crustacean, epigenetics, methylome, intergenerational, genotoxic, pesticides, penoxsulam, epigenotoxicology

## Abstract

Epigenome is susceptible to modulation by environmental pressures—namely, through alterations in global DNA methylation, impacting the organism condition and, ultimately, reverberating on the phenotype of the subsequent generations. Hence, an intergenerational study was conducted, aiming to clarify the influence of genotoxicants on global DNA methylation of the crayfish *Procambarus clarkii*. Two subsequent generations were exposed to the herbicide penoxsulam (Px; 23 µg·L^−1^) and to the genotoxicant model ethyl methanesulfonate (EMS; 5 mg·L^−1^). Px did not induce changes in DNA methylation of adult crayfish (F_0_). However, the hypomethylation occurring in unexposed F_1_ juveniles demonstrated that the history of exposure per se can modulate epigenome. In F_1_ descendants of the Px-exposed group, methylome (hypermethylated) was more affected in males than in females. EMS-induced hypomethylation in adult females (F_0_), also showed gender specificity. In addition, hypomethylation was also observed in the unexposed F_1_ crayfish, indicating an intergenerational epigenetic effect. The modulatory role of past exposure to penoxsulam or to EMS also showed a dependency on the crayfish developmental stage. Overall, this research revealed that indirect experiences (events occurring in a predecessor generation) can have an impact even greater than direct experiences (present events) on the epigenetic dynamics.

## 1. Introduction

Ecotoxicological research has been mostly centered on temporally restricted assessments at individual and sub-individual levels, which can represent a limitation in terms of representativeness, keeping in view the requirement to predict the actual ecological impact of contamination. In this context, the implementation of inter- and transgenerational studies can represent a valuable advance toward the elucidation of processes able to produce deleterious effects at higher organizational levels (e.g., population), thereby increasing the ecological relevance. This approach has been settled mainly through reproductive (e.g., developmental abnormalities, reproductive success) [1] and growth/survival endpoints [2]. More recently and following the conceptualization of epigenetic inheritance [3], the use of epigenetic markers emerged as a novel and promising strategy, offering suitable information on the diagnosis and prediction of ecotoxicological impacts. This is substantiated by the assumption that epigenetic changes can be triggered by environmental factors, such as exposure to contaminants, modulating gene expression, which may have repercussions at the organism and population levels [4]. This embodies an environmental epigenetic perspective, allowing critical progress concerning the knowledge on resistance and adaptation as well as disease and variability processes [5].

DNA methylation was the first epigenetic marker described [6,7] and is still the most studied nowadays in the field of environmental toxicology [8], among a set of parameters also including histone modifications, chromatin remodeling, and non-coding RNA expression (miRNA) [9]. Methylation is the only epigenetic process that directly targets the DNA, where a methyl group replaces the hydrogen atom in the cytosine base, creating thus a new covalent bond [9], whose effects depend on the genome location where it occurs [10]. DNA methylation is involved in many cellular regulation processes, including chromatin condensation, chromosome stability, X-chromosome inactivation, genomic imprinting, and gene transcription [11], playing a crucial role in determining cell normal development, proliferation, and genome stability [9].

DNA methylation is susceptible to environmental pressures, leading to alterations in gene expression [12], being passible to be translated into the whole organism condition and, ultimately, if this epigenetic mark resists erasure waves during embryogenesis [13], could have repercussions on the phenotype of the subsequent generations [8]. Studies addressing the induction of changes in the global DNA methylation by environmental toxicants, encompassing different taxa, were revised by Vandegehuchte and Janssen [4]. For instance, the exposure to Zn induced hypermethylation in the fish *Carassius auratus* [14] and hypomethylation in the crustacean *Daphnia magna* [15].

Another challenging topic in this context is the identification of the concomitant occurrence of events affecting the epigenome and DNA integrity. Sargsyan et al. [16] reported that, in the presence of metals that induce genotoxicity, the lizard *Darevskia armeniaca* also displayed DNA hypomethylation. Moreover, it has been hypothesized that some pesticides, despite not increasing cancer risk directly via a genotoxic process, may operate through epigenetic mechanisms [11].

Most of the studies regarding the modulation of DNA methylation by environmental contaminants were carried out in vertebrates [17,18,19,20,21], and only a few addressed invertebrate species [22,23,24]. Though DNA methylation appears to be associated with gene regulation and expression in both vertebrates and invertebrates [25], their patterns may differ between those animal groups. In the latter, for instance, the genome can have longer sections of methylated DNA interspersed by unmethylated DNA [17]. In particular, the methylated cytosines tend to be part of the gene bodies, while non-coding regions are less methylated [17,25].

While some environmentally induced epigenetic changes are perishable, DNA methylation may be inherited mitotically along with the genetic code from cell to cell (thereby through cell lineage development, persisting during organism’s lifetime [25]), but also meiotically from parent to offspring (intergenerationally) and then to grand-offspring (transgenerationally) [5,8,11,26]. Thus, the convergence of evolutionary developmental biology, environmental toxicology, and epigenetics is particularly important at the earliest stages of development when epigenetic modifications are more vulnerable to perturbation resulting in lifelong and possibly inter/transgenerational effects [26]. One of the first studies on epigenotoxicity of environmental contaminants reported a reduced spermatogenic capacity associated with an abnormal DNA methylation pattern in sperm of rat descendants from breeders exposed to the fungicide vinclozolin [18].

Inherited epigenetic memory can thus determine the responses to a present environmental scenario, either corresponding to repeated exposure (in terms of agent and duration, relative to the precedent generation) or to a new context resulting from the exposure to another agent or to uncontaminated media (possible to occur, for instance, if the habitat was restored or the animal moved to a contamination-free locale).

Therefore, applying an epigenotoxicity approach, this study addressed the methylation patterns in the muscle of *Procambarus clarkii*, intra- and intergenerationally, under present and past scenarios of exposure to the genotoxicants penoxsulam (a new post-emergence herbicide widely used on dry-seeded and water-seeded rice crops in order to control broadleaf weeds, aquatic plants, and certain grasses, approved in the US since 2004 [27] and in the EU since 2010 [28]; its genotoxic potential for aquatic species has already been demonstrated [29,30] including to the *P. clarkii* [31,32]) and ethyl methanesulfonate (EMS, an alkylating agent known for its genotoxic and mutagenic potential on fungi, plants, insects, and human cells [33,34]). The choice of these epigenetic challengers relied on the hypothesis that events affecting DNA integrity may concomitantly affect the epigenome.

The aims of this work were (i) to study the influence of penoxsulam and EMS on DNA methylation of *P. clarkii*, also seeking for gender-related patterns; to pursue an intergenerational approach, evaluating the epigenetic memory in (ii) unexposed crayfish (juveniles and adults) representing the offspring (F_1_) of a genotoxic-exposed generation (F_0_), and in (iii) juvenile crayfish (F_1_) subjected to an exposure corresponding to the same and to a different genotoxicant relative to the stressful scenario experienced by the predecessors (F_0_); (iv) to clarify whether the dynamics of epigenetic changes are determined by direct and indirect (events occurring in the predecessor generation) experiences, thus contributing to the consolidation of an epigenotoxic perspective as a critical element in the ecotoxicology field and risk assessment approaches.

## 2. Materials and Methods

### 2.1. Chemicals

Penoxsulam (Px; CAS No 219714-96-2) and ethyl methanesulfonate (EMS; CAS No 62-50-0) were obtained from Sigma-Aldrich Chemical Company (Madrid, Spain). The NZY tissue gDNA isolation kit (NZYtech) was obtained from NZYtech (Lisbon, Portugal). The MethylflashTM global DNA methylation (5-mC) ELISA Easy Kit (colorimetric) (Epigentek Group Inc.; Farmingdale, NY, USA) was obtained from bioNova científica s.l. (Madrid, Spain).

### 2.2. Animal Maintenance

In order to obtain an initial lot of animals (F_0_), adult crayfish specimens (*Procambarus clarkii*), with an average length of 11.22 ± 0.91 cm, were collected at Minho River (Vila Nova de Cerveira, NW Portugal), a low impacted area concerning the presence of pesticides [35] as well as other inorganic and organic contaminants [36]. In the laboratory, crayfish were kept in individual aquaria for two weeks for acclimation before starting sub-trial 1, with the following water conditions: constant temperature (20 ± 1 °C), fresh water (dechlorinated tap water; salinity 0) with aeration (dissolved oxygen 8.1 ± 0.5 mg·L^−1^), daily UV disinfection, weekly control of nitrites (0.2 ± 0.05 mg·L^−1^), nitrates (25 ± 6.0 mg·L^−1^), ammonia (<0.1 mg·L^−1^), and pH (7.5 ± 0.2). Animals were daily fed ad libitum with crustacean feed, Caridina Vita, produced by Sparos^®^ (Olhão, Portugal).

### 2.3. Experimental Setup

**Figure 1:**
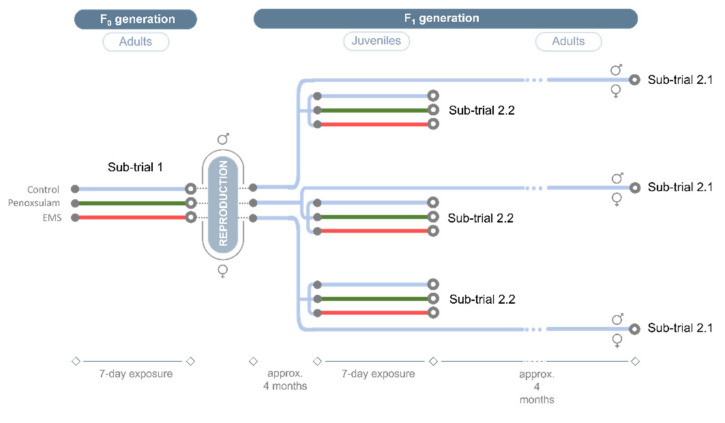
Schematic representation of the experimental design, depicting an intergenerational trial involving the exposure of the red swamp crayfish (*Procambarus clarkii*) to the genotoxicants penoxsulam (Px; green line) and ethyl methanesulfonate (EMS; red line). Sub-trial 1: exposure (7 days) of male (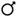) and female (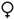) adults, from F_0_, to Px and EMS, in comparison with a control group (blue line). After reproduction of the F_0_ organisms (intragroup crosses), F_1_ offspring were divided into two sub-trials (2.1 and 2.2). Sub-trial 2.1: the progeny (F_1_) of each F_0_ group was allowed to grow in uncontaminated water until adulthood (blue line); global DNA methylation was analyzed in both stages (juvenile and adult). Sub-trial 2.2: the progeny (F_1_) of each F_0_ group was allowed to grow in uncontaminated water only until the juvenile stage and then exposed to Px and EMS for 7 days, and thereafter analyzed in comparison with a control group. 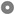 represents the sampling moments.

Figure 1 exhibits the schematic representation of the experimental setup of the intergenerational trial. In sub-trial 1 and in sub-trial 2.2, each crayfish was individually placed in 1 L aquaria to be exposed to a genotoxicant (penoxsulam or EMS), for 7 days. A control group was kept in uncontaminated water. During the exposure period, animals were fed ad libitum in the first 6 days of exposure and fasted the day before sampling. Crayfish of sub-trial 2.1 were maintained in the same conditions as described for the acclimation period (Section 2.2).

The selection of the Px concentration (23 µg·L^−1^) to test relied on its environmental relevance [29], while the EMS concentration (5 mg·L^−1^) was selected according to its use as a positive control on genotoxic assays in fish [37] and crayfish [31].

At the end of each sub-trial, every crayfish was sacrificed with a single cut in the rostrum and the striated muscle (a portion from the ventral–anterior area) was collected (approx. 1 g) and preserved in ethanol absolute until epigenetic analysis.

Sub-trial 1: direct exposure of adult crayfish (F_0_)

Sub-trial 1 aimed to explore the influence of penoxsulam and EMS on DNA methylation of adult *P. clarkii*, from F_0_, also seeking gender-related patterns. Therefore, adult crayfish, forming experimental groups of 14 animals (*n* = 7 of each gender), were individually exposed to 23 µg·L^−1^ of Px or to 5 mg·L^−1^ of EMS, for 7 days (Figure 1), in 1 L aquaria (water and room conditions were the same as the acclimation period; Section 2.2). A control group (*n* = 7 of each gender; C) was maintained in uncontaminated freshwater. Genotoxic and control media were daily renewed. Afterward, animals were sacrificed, and muscle samples were extracted as described above.

Sub-trial 2.1: indirect exposure of juvenile and adult crayfish (F_1_)

This sub-trial was established to pursue an intergenerational approach, evaluating the epigenetic memory in unexposed juvenile and adult crayfish from F_1_, representing the offspring of a genotoxic-exposed generation (F_0_).

Following the exposure described in sub-trial 1, crayfish (F_0_) were paired and allowed to reproduce (intragroup crosses). During the mating period, the temperature was set for 24.0 ± 1.0 °C. Females with eggs were relocated in individual aquaria, and the water temperature was maintained. After hatching, juveniles (F_1_) were transferred to a new aquarium (offspring were separated according to the provenience group F_0_; Figure 1) to grow in uncontaminated water until reach the adult stage. The freshwater medium was weekly renewed; temperature and water conditions were similar to the acclimation period (see Section 2.2). This sub-trial had two sampling moments: the first, when juveniles reached 4 months of age (*n* = 6, from each independent group; the average length of 4.02 ± 0.17 cm), and the second when adult crayfish reached 8 months old (*n* = 4; 2 of each gender, from each independent group, with an average length of 7.35 ± 0.16 cm). In each sampling moment, every crayfish was sacrificed, and muscle samples were extracted as described above.

Sub-trial 2.2: direct exposure of juvenile crayfish (F_1_) under the influence of F_0_ (indirect) exposure

This sub-trial aimed to pursue an intergenerational approach to evaluate the epigenetic memory in juvenile crayfish (F_1_) subjected to an exposure corresponding to the same or to a different genotoxicant, relative to the stressful scenario experienced by the previous generation (F_0_). Due to sexual immaturity, no gender discrimination was carried out. Briefly, F_1_ 4-month-old juveniles (with an average length of 4.02 ± 0.17 cm; *n* = 54; 6 (animals) × 3 (treatment) × 3 (F_0_ groups)) descendants from the F_0_ crossings (i.e., males mated with females both exposed to Px; males mated with females both exposed to EMS; unexposed males mated with unexposed females) were exposed to 23 µg·L^−1^ of Px, 5 mg·L^−1^ of EMS and uncontaminated fresh water for 7 days, at 20 ± 1.0 °C (Figure 1). Genotoxic and control media were daily renewed. In the end, animals were sacrificed, and muscle samples were extracted as described above.

### 2.4. DNA Extraction

DNA from each muscle sample was extracted using the NZY tissue gDNA isolation kit, according to the manufacturer’s instructions. Briefly, a 20 mg piece of tissue was cut into small pieces and placed overnight in the microcentrifuge tube at 56 °C with proteinase K and buffer solution (NT1; kit component). After this incubation, 200 µL of lysis solution were added to each sample, and the mixture was vortexed for 10 s. Next, 210 µL of ethanol absolute were added and the mixture immediately vortexed. The mixture was transferred into an NZYSpin tissue column, placed in a 2 mL collection tube, and centrifuged for 1 min at 11.000 g. Then, the silica membrane of the NZYSpin tissue column was washed and dried. Thereafter, each DNA sample was eluted with 60 µL of sterile distilled water (at 70 °C). The genomic DNA was then stored at 4 °C, until further analysis.

### 2.5. DNA Methylation Analysis

**Figure 2:**
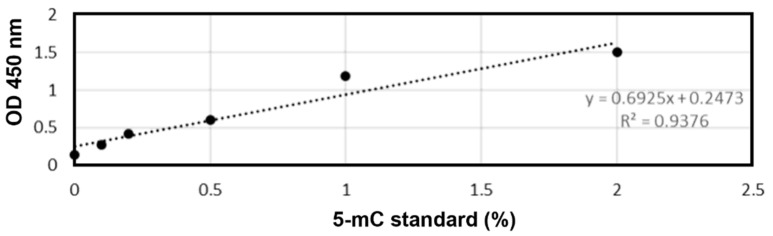
Standard curve for methylated DNA, built using the kit-provided positive controls for validation of the measurement method (ELISA Easy Kit EpiGentek).

The global DNA methylation of each sample was quantified using MethylflashTM global DNA methylation (5-mC) ELISA easy colorimetric kit, in accordance with manufacturer’s instructions. Briefly, 100 µL of a binding solution were added to each well (of a 96-well plate), followed by 2 µL of DNA sample (samples were diluted to obtain 100 ng in a volume of approximately 2 µL, as suggested by the manufacturer’s protocol instructions). Additionally, negative (NC) and positive (PC) controls were considered to the plate to generate the standard curve (as represented in Figure 2).

Samples were incubated at 37 °C, for 60 min. Then, each well was washed three times, and samples were incubated with 50 µL of the 5 mC detection complex solution for 50 min. Each well was rewashed five times, and samples were incubated with 100 µL of developer solution for 3 min. The developer solution turned blue in the presence of sufficient methylated DNA. The color in the NC wells remained unchanged. When the PC samples became blue (indicating the presence of methylated DNA), the enzyme reaction was stopped with the stop solution, and the color of each sample changed to yellow. The absorbance was immediately read at 450 nm.

### 2.6. Statistical Analysis

Statistical analysis was performed with the software Statistica 7.0. Data were first tested for normality and homogeneity of variance to meet statistical demands by Shapiro–Wilk’s W test and Brown–Forsythe (HOV) test, respectively, to apply parametric tests. Two-way ANOVA (gender x treatment), followed by Fisher’s LSH post hoc test, was used to compare F_0_ adults (sub-trial 1). Males vs. females, within each treatment, were compared using a *t*-test. A one-way ANOVA, followed by Dunnet’s post hoc test, was used to compare the F_1_ juveniles of sub-trial 2.1. Additionally, a one-way ANOVA, followed by Fisher’s LSH post hoc test, was used to compare F_1_ adults of the same gender (sub-trial 2.1). A *t*-test was used to compare genders within the same treatments of F_1_ adults. Another two-way ANOVA (history × treatment), followed by Fisher’s LSH post hoc test, was used to compare F_1_ juveniles from treated groups with the corresponding group with different history profiles (the exposure in the F_0_ group corresponds to a past; history) exposure in F_1_ (sub-trial 2.2). Differences between groups were considered significant when *p* < 0.05 [38].

## 3. Results

### 3.1. Sub-Trial 1: Direct Exposure of Adult Crayfish (F_0_)

**Figure 3:**
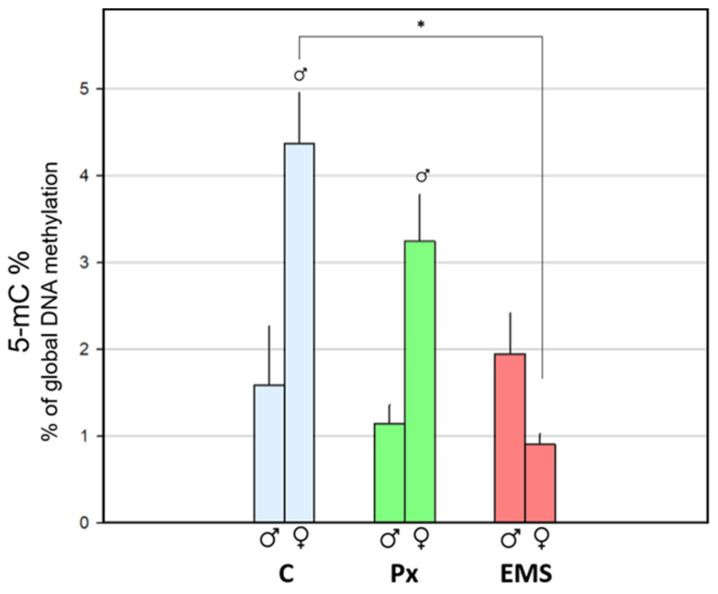
Sub-trial 1—Global DNA methylation measured in muscle of F_0_ *Procambarus clarkii*, both adult males (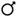) and females (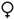), following exposure to 23 µg·L^−1^ of penoxsulam (Px; green) or 5 mg·L^−1^ of ethyl methanesulfonate (EMS; red), in comparison with the corresponding control groups (C; blue). Bars represent the standard error. Statistically significant differences (*p* < 0.05) are: (*) between treatments, within the same gender; (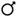) vs. male group, within the same treatment.

Males’ global methylation (5 mC content) presented a similar pattern in all treatments, and no significant differences were observed in the exposed groups with respect to the control (C) (Figure 3). Regarding females, the 5 mC levels in the group exposed to EMS were significantly lower (4.82-fold; *p* < 0.001) in relation to the unexposed group (C), while no significant differences were observed between crayfish exposed to Px and C (Figure 3).

The females from C and Px groups displayed higher 5 mC content when compared with the respective male groups (*p* = 0.009 and *p* = 0.006, respectively) (Figure 3). In the group exposed to EMS, despite the absence of statistical differences, males showed a tendency of higher 5 mC levels (2.14-fold) in relation to females.

### 3.2. Sub-Trial 2.1: Indirect Exposure of Juveniles and Adult Crayfish (F_1_)

**Figure 4:**
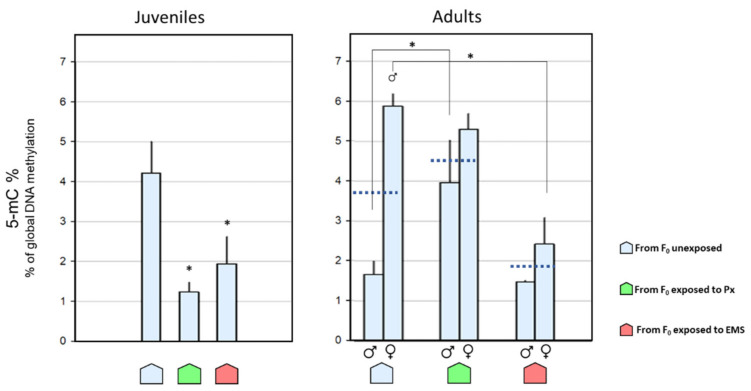
1—Global DNA methylation measured in muscle of unexposed F_1_ *Procambarus clarkii*, in juveniles (left) and adults (right; males: 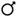; females: 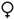), descendants from F_0_ unexposed (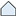), penoxsulam-exposed (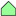), and ethyl methanesulfonate-exposed (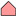) groups. Dark blue dashed lines represent the 5-mC% mean values for the corresponding adult groups combining both genders. Bars represent the standard error. Statistically significant differences (*p* < 0.05) are: (*) vs. descendants from F_0_ unexposed group; (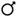) vs. male group, within the same exposure background.

Concerning the juvenile stage, data indicated a significantly lower DNA methylation in the groups descending from F_0_ exposed groups (both Px and EMS) when compared with the offspring of the unexposed group. Specifically, 3.40-fold (*p* = 0.002) and 2.17-fold (*p* = 0.02) lower 5 mC levels were detected in descendants from the Px-exposed and EMS-exposed groups, respectively, in comparison with the offspring of the unexposed group (Figure 4).

Adult males descending from the Px group presented significantly higher (2.38-fold; *p* = 0.03) 5 mC levels than those descending from the unexposed group (Figure 4). Regarding females, crayfish descending from the EMS group presented significantly lower global DNA methylation (2.42-fold; *p* < 0.001) when compared with offspring of the unexposed group (Figure 4).

Comparing genders, females descending from the unexposed group presented higher 5 mC levels of (3.5-fold; *p* < 0.001) than the corresponding males. The crayfish descendants from EMS-exposed groups did not present any differences between genders (Figure 4).

Contrarily to juveniles (sexually immature [39,40]), the quantification of DNA global methylation in adult crayfish is considered gender separation. In Figure 4, dark blue dashed lines represented correspond to the 5 mC mean values for each adult group combining both genders. It is visible in all groups that the % global methylation in females is higher than the average of the two genders together, and, on the other hand, the 5 mC level in males is lower than the determined average.

### 3.3. Sub-Trial 2.2: Direct Exposure of Juvenile Crayfish (F_1_) under the (Indirect) Influence of F_0_ Exposure

**Figure 5:**
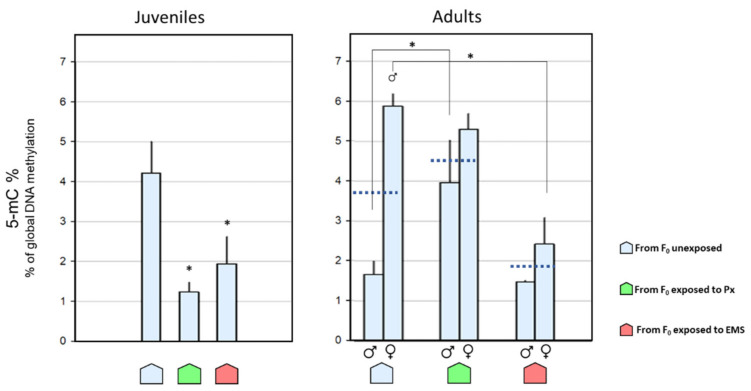
Sub-trial 2.2—Global DNA methylation measured in muscle of F_1_ juvenile *Procambarus clarkii*. Descendants from F_0_ unexposed (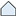), penoxsulam-exposed (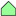), and ethyl methanesulfonate-exposed (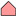) groups were currently exposed to 23 µg·L^−1^ of penoxsulam (Px; green) or to 5 mg·L^−1^ of EMS (EMS; red) and compared with the control groups (C; light blue; it should be noted that these data are also represented in Figure 4). Bars represent the standard error. Statistically significant differences (*p* < 0.05) are (*) between different historical backgrounds, within the same current treatment.

No significant differences were detected in global DNA methylation when the experimental groups (C, Px, and EMS) were compared within the same exposure history (Figure 5).

On the other hand, significant differences were detected when comparing different historical backgrounds within the same current treatment. Globally, it was discernible a variation pattern with lower global DNA methylation in crayfish descendants from Px- and EMS-exposed groups in comparison with those descending from unexposed parents. Specifically, juveniles with a history of exposure to penoxsulam showed a decrease in the percentage of methylated cytosines of 2.14-fold (*p* = 0.04), when exposed to penoxsulam, and 3.59-fold (*p* = 0.003) when exposed to EMS, compared with juveniles exposed to the same compounds but with a history of no contamination. On the other hand, juveniles with a history of exposure to EMS showed an increase in methylated cytosines when exposed to EMS, compared with juveniles with a history of penoxsulam (2.86-fold; *p* = 0.02) also exposed to this compound (Figure 5).

## 4. Discussion

This work aimed to understand how two different genotoxicants (Px and EMS) affect the DNA methylation pattern in both genders of the species *Procambarus clarkii* and to what extent the methylation pattern of a given offspring is influenced by the genotoxic exposure of the progenitors, contributing to predict the ecological impact of the complex interactions of past and present exposures affecting wild populations under real field scenarios.

*P. clarkii* can adapt very quickly to environmental stressors, presenting also high tolerance to environmental heterogeneity (e.g., regular periods of drought, omnivorous diet) [41], features that contribute to its success as an invasive species. These characteristics may also justify why this species can be found in inhospitable environments, such as those impacted by pesticides (e.g., rice fields) [41]. Since *P. clarkii* reaches maturity maintaining a small body size, has a rapid growth rate, a large number of offspring, and a relatively short life span [42], is regarded as a suitable non-model organism for intergenerational studies. To date, there are no studies regarding DNA methylation in *P. clarkii*, though Vogt [43] assessed this epigenetic process in the muscle of the congener species *P. fallax*, the marbled crayfish. Thus, global DNA methylation was the marker chosen to study in the epigenome of the F_0_ and F_1_ generation of *P. clarkii*, within the framework above enunciated.

### 4.1. DNA Methylation in P. clarkii (F_0_) after Direct Genotoxic Exposure

The methylation pattern can be altered by external factors, such as the presence of contaminants [14,44]. A study with *D. magna* showed that exposure to the fungicide vinclozolin [16] induced hypomethylation, which may affect gene regulation and expression. In the present study, no changes were observed in the DNA global methylation following direct exposure to the herbicide penoxsulam, in both genders. In the work developed by Akcha et al. [45], it was demonstrated a positive correlation between the presence of oxidative DNA damage, as measured by the level of 8-oxodGuo, and DNA methylation, as measured by human DNMT1 (DNA methyltransferase involved in methylation maintenance) activity. In line with these findings, it can be inferred that penoxsulam, probably, has a negligible pro-oxidant potential, which was corroborated in *P. clarkii* spermatozoa by Marçal et al. [32].

Under exposure to EMS, the DNA of female crayfish became hypomethylated. This could be supported by the fact that EMS induces base replacements of guanine–cytosine (C/G) to adenine–thymine (A/T) [46]. Therefore, once EMS may reduce the cytosines, the amount of 5 mC may also be diminished. However, global DNA methylation in male crayfish was not diminished by the EMS, which could be related to a naturally less methylated epigenome in the striated muscle cells of this gender (as recurrently observed in the different components of the current study), limiting the margin for a reduction. To the authors’ knowledge, this study provides the first results regarding EMS effects on the crayfish epigenome.

Small differences in global DNA methylation can have great consequences for the phenotype [40]. In an in vitro study performed by Hiendleder et al. [47] with bovine fetuses, phenotypic features as fetal overgrowth and endocrine changes were related to only 11.2% deviation from normal methylation values in the liver. In the present study, females exposed to EMS presented a 60 % deviation from the normal/basal global DNA methylation profile. Although it was not the focus of this study, considering that the deviation currently described is almost 6 times that reported by Hiendleder et al. [47], important phenotypic changes in crayfish can be hypothesized due to this shift in the global methylation. Nevertheless, it is not expectable that the measured changes were fully and proportionally mirrored in the loss of DNA homeostasis and genomic instability. Future studies are required in this direction.

Gender is an important variable to consider when assessing global DNA methylation. In this study, it was disclosed, for the first time, the global methylation basal values in the striated muscle of the crayfish *P. clarkii* with gender discrimination. Specifically, males presented less methylated cytosines than females. Considering previous research with invertebrates, and particularly with the insect *Acyrthosiphon pisum* [48] and the crustacean *D. pullex* [49], it was described that males presented higher levels of global DNA methylation than females. Contrary to the present study, in which there was only one tissue analyzed, the striated muscle, the studies mentioned above [48,49] analyzed the methylome in the whole body. Therefore, it can be only concluded that males of *A. pisum* and *D. pullex* have globally more methylated cytosines than females; the tissue-specific ratio of methylated cytosines for each gender remains unknown. Therefore, regarding the current results, gender-dependent differences in adult crayfish epigenome suggest a higher basal global DNA methylation on the muscle of females, which represents an innovative aspect. To the authors’ knowledge, no solid scientific information is available on the mechanistic understanding of gender specificity—namely, applicable to DNA methylation in crustaceans.

Gender-related methylation patterns have been reported also in ecotoxicological studies (mostly in vertebrates) in association with contaminants exposure. It has been reported that compounds with the ability to modulate DNA methylation may affect differently males and females. For instance, male zebrafish presented greater changes in the DNA methylation patterns in the brain and eyes, after chronic exposure to depleted uranium, than females [50]; moreover, males polar bear presented their DNA methylation in the brain more affected than females after mercury exposure [21].

### 4.2. DNA Methylation in Unexposed Crayfish Descendants (F_1_) from a Genotoxic-Exposed Generation (F_0_)

The history of exposure to penoxsulam showed to have an impact on the global methylated cytosines of the offspring (F_1_). The F_1_ young crayfish grown in an uncontaminated environment showed a decrease in 5 mC content (DNA hypomethylation). DNA hypomethylation is the most consistent epigenetic alteration observed in cancer studies [51], while genes involved in the development, tissue-specific functions, or response to environmental stimuli are poorly methylated and could be associated with higher phenotypic plasticity [45]. Therefore, although not being possible to identify the consequences for the F_1_ generation from the contact to penoxsulam in the previous generation, the modulating effect on the DNA methylation in the offspring was evident, highlighting an intergenerational impact of penoxsulam. Interestingly, it should be recalled that the direct exposure to penoxsulam (F_0_ generation) did not alter DNA methylation (see Figure 3). In agreement, though testing a different agent, a study with *D. magna* reported that the epigenetic effects of Zn were only observed in the F_1_ generation, where the global DNA methylation was also diminished [15]. These authors suggested that Zn reduced the substrate for DNA methylation since the concentration of metallothioneins (which interacts with homocysteine to form conjugates) increased after the exposure; this conjugation caused a decrease in the availability of free homocysteine, used as a substrate to form methionine (which is converted to S-adenosyl-methionine, an important methyl donor for DNA methyltransferases) [15]. Furthermore, when detoxification processes are favored, homocysteine, which could be also needed for DNA methylation, is exploited for glutathione synthesis [52].

Adult males (F_1_) showed a significant increase in the global DNA methylation (hypermethylation) in relation to those descending from the unexposed group. DNA hypermethylation is considered the default epigenetic state and serves in maintaining genome integrity [53]. The DNA hypomethylation observed before in F_1_ juveniles, along with the hypermethylation observed in adult F_1_ males, reinforces the suggestion that penoxsulam can change the DNA methylation pattern across generations. Studies with the European honeybee *Apis melifera* [54,55] reported that genes predicted to be hypermethylated are associated with housekeeping functions, while those predicted to be hypomethylated are associated with general immune functions. Hypermethylation of intragenic regions of housekeeping genes is consistent in the invertebrate species *Crassostrea gigas* and *A. mellifera* [23,54]. Therefore, the present data concerning muscle give plausibility to the hypothesis that penoxsulam can induce different phenotypic changes depending on the developmental stage. In future studies, it will be important to considerer the evaluation of gene expression, since it highlights the genes that could be vital to cell metabolism, including pathways related to the immune system.

The data obtained for the descendants from the F_0_ Px-exposed group show that the epigenome of females (adults) did not seem to be affected by penoxsulam. A similar pattern (i.e., the percentage global methylation of Px-exposed females similar to the unexposed females) was also observed on their offspring F_1_ (i.e., females F_1_ Px-exposed descendants presented a percentage of methylated cytosines similar to females F_1_ unexposed descendants). Hence, it was demonstrated that penoxsulam induced changes indirectly in the crayfish offspring epigenome, in addition to having a gender-specific intergenerational epigenetic effect.

As observed in the descendants from the F_0_ Px-exposed group, the history of exposure to EMS showed to have an impact on the juveniles grown in an uncontaminated medium, where DNA hypomethylation was observed. In adults, the EMS intergenerational effect in unexposed male crayfish was not evident, contrary to what was observed in Px-descendant males. However, the hypomethylation observed in the unexposed F_1_ females descended from the EMS-exposed group, representing a similar pattern to the F_0_ females exposed to EMS, suggests that F_1_ females had an epigenetic memory (i.e., it was observed a hypomethylation similar to in the progenitors) of the F_0_ exposure. Accordingly, in a study with *D. magna*, an exposure to 5-azacytidine (a DNA methyltransferases inhibitor) induced epigenetic changes in the F_0_ generation, which were transmitted to the unexposed F_1_ and F_2_ generations [16].

The current outcomes suggest that EMS had a potential gender-specific intergenerational epigenetic effect. Moreover, the exposure to this genotoxicant affected the females’ methylome (since F_0_ generation) more, contrary to what was observed with penoxsulam exposure (in this study) as well as with uranium [50] and mercury [21] exposures. This highlights gender as an important variable that cannot be overlooked when studying this type of parameter in sexually mature animals.

The outcomes of this section—namely, that animals displayed epigenetic changes, despite only having contact with the genotoxicants in the previous generation (indirect exposure), confirmed the importance of the incorporation of the first exposed generation and the subsequent generations on risk assessment [8].

### 4.3. DNA Methylation in Juvenile Crayfish (F_1_) Submitted to a Current Exposure

#### 4.3.1. Exposure to the Same Genotoxicant

The juvenile crayfish (F_1_) were subject to an exposure corresponding to known genotoxicants (i.e., the same genotoxic agent experienced by the predecessors). From these data, it was possible to understand that the exposure to Px in the F_0_ generation greatly influenced the methylation pattern on the next generation (F_1_). When juveniles, descendants from the F_0_ Px-exposed group, were exposed to penoxsulam, they presented a hypomethylation, compared with the crayfish that was in contact with penoxsulam for the first time. This eventual epigenetic memory, transmitted by the progenitors, was also observed in the unexposed juveniles, descendants from the F_0_ Px-exposed group. Since this group did not contact directly with penoxsulam, this outcome supports the potential of this herbicide to induce generational epigenetic changes, specifically on DNA methylation. To the authors’ knowledge, there are no scientific studies addressing this aspect, i.e., what happens to the methylation pattern of the offspring (with a history of exposure from the previous generation) when facing exposure to a genotoxicant, since most research focuses on the effects caused by parental exposure on subsequent unexposed generations.

Considering the descendants from the F_0_ EMS-exposed group, F_1_ juveniles appeared to have also inherited the memory of their parents’ exposure. The similarity of DNA methylation profile between the unexposed F_1_ crayfish and the F_1_ EMS-exposed suggests that a tolerance to this compound may have been acquired. It should also be noted that, while the F_0_ EMS-exposed groups had less methylated cytosines (in both crayfish genders) when compared with the unexposed group, their descendants after being exposed to the same compound, i.e., EMS, tended to display higher DNA methylation than the unexposed group (despite without statistical significance). This reinforces the theory that the memory of the genotoxic exposure in F_0_ was transmitted to F_1_, strengthening the stability of methylation processes when F_1_ is exposed to the same genotoxicant as in F_0_. 

#### 4.3.2. Exposure to a Different Genotoxicant

Juveniles (F_1_) were exposed to a different genotoxicant (i.e., distinct from that experienced by the ancestors). The decrease in % of methylated DNA in the unexposed F_1_ crayfish derived from F_0_ genotoxic-exposed groups (Px and EMS F_0_ groups) revealed that the offspring suffered an indirect impact from the genotoxic pressure in the F_0_ generation. These results are particularly important since there is a lack of scientific information elucidating what happens at the level of DNA methylation when offspring are subjected to a new genotoxic exposure. Oppold et al. [52] exposed an F_0_ generation of the Asian tiger mosquito (*Aedes albopictus*) to vinclozolin (fungicide) and observed a decrease in the offspring (F_1_) sensitivity to the insecticide imidacloprid (hypomethylation was observed). Their results suggest that the epigenetic marker DNA methylation may be involved in the mechanisms that allow adaptation (e.g., lower vulnerability) to insecticides [52]. Although it remains to be elucidated how organisms acquire toxic resistance, Bates et al. [56] reported that low doses generally provide the best opportunity for its development. It is important to recall here that the species used in the present study (*P. clarkii*) is known for being an invasive species in European, African, and Asian ecosystems, with successful physiological strategies (phenotypic characteristics) even in inhospitable environments. This raises a key question of whether the observed changes in methylome due to exposure to an environmentally relevant concentration of penoxsulam, specifically in juveniles and male adults, may be behind an adaptive strategy for this species.

Juvenile crayfish from the F_0_ Px-exposed group presented hypomethylation after exposure to EMS, indicating that, in the presence of a different genotoxic challenge, the memory of F_0_ does not seem to prevent global methylation changes. In contrast, the % of DNA methylation increased in Px-exposed juveniles descended from EMS-exposed F_0_, which may indicate the emergence of mechanisms that will permit the exposed organisms to tolerate the stress and survive [57].

Again, without data regarding gene expression information (i.e., what genes in the crayfish are over- or underexpressed due to penoxsulam and EMS exposure), it is not possible to understand if the induced epigenetic inheritance was a burden or a gift. Therefore, in future work, in addition to an analysis of the gene expression being a noteworthy prospect, it will also be of interest to see what phenotypic changes are occurring, for example, from possible shifts in metabolic pathways to changes in reproductive and morphological features. It is noticeable, however, that these compounds induced changes in the methylation pattern, and this probably had consequences for the organisms and, consequently, for their populations.

### 4.4. The Legacy of a Parental Exposure: An Overview

Epigenetic transmission enables parents to influence the phenotypes of their offspring, thus providing a mechanism by which the parental environment can influence the offspring’s performance [58].

The impact of parental exposure to penoxsulam on offspring appears to vary with the crayfish stage of development. Accordingly, juveniles (F_1_) presented hypomethylation, whereas hypermethylation occurred in the sexual maturity phase (adult males of F_1_). This represents further evidence that methylome is not stable throughout the life cycle.

This study showed that, in the juvenile stage, when crayfish face a new exposure to the same (penoxsulam) or to a different genotoxicant (EMS), no significant changes were observed in the level of methylated cytosines (in relation to the unexposed crayfish also with historical exposure to penoxsulam). However, comparing the offspring resulting from the herbicide-exposed group with the offspring from the non-exposed group, it was possible to perceive that, in general, penoxsulam induced a strong decrease in juveniles’ global methylation.

Concerning the impact on offspring of parental exposure to EMS, it was pointed out an identical pattern (when compared with penoxsulam groups). Thus, the unexposed offspring presented hypomethylation in juveniles (F_1_) and hypomethylation in adults females (F_1_). When offspring from the EMS-exposed group were compared with the non-exposed group, it was shown that the parental exposure to EMS also induced a decrease in global methylation, but only considering the unexposed juveniles. Moreover, juveniles exposed both to the same (EMS) and to a different (penoxsulam) genotoxicant did not have their global DNA methylation changed. Contrary to what happened in EMS-exposed F_0_ adults (both genders), the level of methylated cytosines in F_1_ juveniles did not decrease following exposure to EMS. Again, this could indicate that the historical impact of the parental exposure to EMS may provide to juvenile specimens some mechanisms to better tolerate stress and survive.

Bearing all this in mind, and considering future research in this thematic, it will be important to address the intergenerational effects when only one parent is exposed (e.g., F_0_ exposed male × F_0_ unexposed females and vice versa). Moreover, it would be also interesting to consider the different organism’s tissues/organs, as it has been reported that different cell types respond differently to the modulating action of xenobiotics on DNA methylation (e.g., Akcha et al. [45]). Akcha et al. [45] observed that the epigenetic effect of diuron (an herbicide) seemed to be tissue specific in *C. gigas*, where DNA hypermethylation occurred in the digestive gland but not in gills and gonads. Therefore, it will be important to investigate in future studies which tissues other than muscle may be susceptible to penoxsulam-induced changes on global DNA methylation. In fact, in future works, it would be particularly interesting to study the penoxsulam effect on the methylome of germ cells since it is this epigenome that will be transmitted to the offspring.

Epigenetic changes, including in DNA methylation, constitute the basis for long-term adaptations [59]. In line with this finding, the present study suggests that the modulation of epigenome may partially explain the *P. clarkii* success as invasive alien species [60,61], “conquering the world” and inhabiting from pristine to highly impacted environments. Overall, current data confirmed the occurrence of intergenerational epigenetic memory, evincing that the consequences of a given exposure to environmental stressors are not confined to the respective generation, which, using a grammatical analogy, can be translated into a conjugation in past continuous rather than past simple.

## 5. Conclusions

The present findings demonstrated, for the first time, the presence of DNA methylation in the species *Procambarus clarkii*, specifically in the striated muscle. Moreover, it was demonstrated that the global DNA methylation in this tissue differs naturally between genders, with females showing higher levels.

The herbicide penoxsulam did not induce changes in DNA methylation of adult crayfish (F_0_). However, the hypomethylation occurring in unexposed F_1_ juveniles demonstrated that the history of exposure per se (indirect exposure) can modulate epigenome. In the F_1_ descendants of the penoxsulam-exposed group, males’ methylome (hypermethylated) was more affected than in females, showing gender specificity.

The genotoxicant model EMS induced hypomethylation in *P. clarkii* adult females (F_0_), also showing to be gender specific. In addition, hypomethylation was also observed in the unexposed F_1_ crayfish, revealing an intergenerational epigenetic effect.

The modulatory role of the historic exposure to penoxsulam or to EMS showed also a dependency on the crayfish developmental stage.

Overall, this work revealed that indirect experiences (events occurring in the predecessor generation) can have an impact even greater than direct experiences (present events) on the epigenetic dynamics.

Finally, it is strongly recommended to consider epigenotoxic approaches as a critical element to thoroughly identify hazards and risk factors associated with environmental contaminants.

## Data Availability

Not applicable.
